# Nutritional quality of beverages available in vending machines in health and social care institutions: do we really want such offers?

**DOI:** 10.1186/s41043-021-00250-1

**Published:** 2021-07-02

**Authors:** Urška Rozman, Nataša Fidler Mis, Urška Pivk Kupirovič, Igor Pravst, Primož Kocbek, Maja Strauss, Sonja Šostar Turk

**Affiliations:** 1 Faculty of Health Sciences, University of Maribor, Žitna ulica 15, 2000 Maribor, Slovenia; 2 Department of Gastroenterology, Hepatology and Nutrition, University Children’s Hospital, University Medical Centre Ljubljana, Bohoričeva 20, 1000 Ljubljana, Slovenia; 3 Nutrition Institute, Tržaška cesta 40, 1000 Ljubljana, Slovenia

**Keywords:** Beverages, Vending machines, Social care institutions, Health care institutions, Slovenia

## Abstract

**Background:**

Vending machines represent one way of offering food, but they are overlooked in the efforts to improve people’s eating habits. The aim of our study was to analyse the variety and nutritional values of beverages offered in vending machines in social and health care institution in Slovenia.

**Methods:**

The available beverages were quantitatively assessed using traffic light profiling and the model for nutrient profiling used by Food Standards Australia New Zealand. Vending machines in 188 institutions were surveyed, resulting in 3046 different beverages consisting of 162 unique product labels.

**Results:**

Between 51 and 54% of beverages were categorised as unhealthy with regard to sugar content. Water accounted for only 13.7% of all beverages in vending machines. About 82% of beverages in vending machines were devoted to sugar-sweetened beverages, the majority (58.9%) presented in 500-ml bottles. The average sugar content and average calories in beverages sold in vending machines are slightly lower than in beverages sold in food stores.

**Conclusions:**

We suggest that regulatory guidelines should be included in the tender conditions for vending machines in health and social care institutions, to ensure healthy food and beverage choices.

## Background

Vending machines usually carry snacks and beverages of low nutritional value, but high in calories, fats, salt and sugar [1, 2], whereas healthy options are hardly offered or totally absent [3, 4]. Considering that such options are available in social and health care institutions, unhealthy dietary choices are available to medical and hospital personnel working long hours, patients, residents and visitors. This is contrary to the World Health Organization (WHO) vision of hospitals that should contribute to building a stronger health system and a healthy community [5]. As hospitals matter to people [5], should they not be a model of offering healthy dietary choices in their vending machines? If alcohol and tobacco products are not sold in health care institutions, why do they offer unhealthy dietary choices?

Beverages with added free sugars represent a major share of the available beverages in vending machines [6, 7]. Several systematic reviews and meta-analyses of cohort studies and randomized clinical studies demonstrated that drinking sugar-sweetened beverages (SSB) increases not only the risk for weight gain and obesity, but also the risk for tooth decay (caries), type 2 diabetes mellitus, cardiovascular diseases, non-alcoholic fatty liver disease, impaired nutrition and several other adverse health effects [8–15]. Especially worrisome are obesity and type 2 diabetes, as they emerged as epidemics of modern societies [16, 17]. On the global level, WHO tackles this problem in the Action Plan for the Prevention and Control of Non-Communicable Diseases in the WHO European Region 2016–2025 [18], whereas on the local level, Slovenia’s National Assembly adopted the “National Program on Nutrition and Health Enhancing Physical Activity 2015–2025” which is coordinated by the Ministry of Health. One of its ten priority areas is the role of health care for maintaining health and for preventing chronic diseases and obesity. Proper nutrition, physical activity and healthy diet choices provided to patients, staff and visitors in health and social care institutions are emphasised as a prerequisite for successful treatment [19].

Increasing numbers of studies show that a well-designed graphic, i.e. traffic light labelling, on the front of food packages, can provide consumers with quick and clear summarised information on nutritional quality and help them to compare products to make healthier choices. Traffic light labelling was first introduced by the Food Standards Agency in the UK in 2007, but the threshold values for categories have changed multiple times since then [20]. There is currently no international consensus about the broadly acceptable standardized nutritional profile model that could be used for front-of-pack summaries of the nutritional quality of foods. Australia and New Zealand show best practise in policy implementation in this area that could be followed by other countries. They use the Food Standards Australia New Zealand (FSANZ) Nutrient Profiling Scoring Criterion model [21] to determine the eligibility of foods and beverages to carry health claims, meaning that only foods with better nutritional quality are allowed to carry health claims. A recent study in Slovenia [22] also evaluated the nutritional quality in food supplies based on the FSANZ model and found that 50% of available beverages in food stores are of lower nutritional quality. The aim of our study was to analyse the variety and nutritional quality of beverages available in vending machines placed in social and health care institution in Slovenia. The resulting guidelines for providing healthier choices for hospital workers, patients and visitors could also support future regulatory interventions.

## Methods

### Sample

Our sample consisted of vending machine displays in all Slovenian hospitals (26), community health centres (64) and all but one nursing homes (98 out of 99), with one exception due to withheld data. The survey was carried out between June and August 2018. All accessible vending machines in each facility were surveyed. The collected data was based on face-front items, i.e. the item in the vending machine slot that was next in line to be sold [1]. If there were five or more empty places of products in a vending machine at the time of the survey, the data collection was repeated on another occasion. Vending machine displays in 188 institutions resulted in 5625 face-front items, with beverages accounting for 54.2% (n=3046) items and consisting of 162 unique product labels.

### Product categorisation

Each beverage was categorised based on the content of sweeteners, and further subcategorised according to the type of beverage [23] (Table 1).

**Table 1:** Beverage categorisation

Category	N (%)	Subcategory
Soft drinks	2286 (75.0)	Carbonated beverages with sugar, carbonated drinks with no sugar and added aspartame/acesulfame-k sweetener, non-alcoholic beer, non-carbonated beverages with sugar, sugar-tasting water, water with taste but no sugar
Waters	417 (13.7)	Mineral water, water
Coffee	119 (3.9)	Iced coffee
Juices	113 (3.7)	100% natural juice, smoothie
Energy drinks	111 (3.6)	Energy drinks with no sugar and added aspartame/acesulfame-k sweetener, energy drinks with sugar

### Nutrient profiling

For each product, nutritional data labelled on beverages, such as fat, saturated fatty acids, sugars, sodium, salt, protein, fibre, energy value and proportion of fruits and vegetables stated in the ingredient list were used. Each product was categorised according to average fat, saturated fatty acid, sugar, and salt values per 100 ml with dietary traffic light labelling (Table 2) as a quality indicator. In the current survey, we used the threshold values listed in Table 2, which are implemented in Slovenia in a variety of programmes for facilitating healthy food choices, such as in the government-funded smart mobile application VesKajJes [24] and the purpose of examining the displays in vending machines at selected Slovenian faculties by the Consumer Association of Slovenia. Green means low content of critical nutrients, while red indicates high content of such constituents [25].

**Table 2:** Criteria for traffic light labelling of beverages in Slovenia

Quality indicator	Green	Amber	Red
	[g/100 mL]		
Fat	< 1.5	1.5–10	> 10
Saturated fatty acids	< 0.75	0.75–2.5	> 2.5
Sugar	< 2.5	2.5–6.3	> 6.3
Salt	< 0.3	0.3–1.5	> 1.5

To compare the nutritional quality of the displayed items in vending machines to the displays in stores with the Food Standards Australia New Zealand (FSANZ) [21], the Nutrient Profiling Scoring Criterion FSANZ model was used. This is a scoring model; foods receive positive and negative points for energy, key nutrients and constituents, and there are different (sum) score thresholds for three types of products (beverages, foods and fats and cheese with high calcium content). For this study, foods that did not pass the FSANZ criterion were considered as less healthy. A similar approach was used in a recent assessment of the nutritional quality of foods available in Slovenian food supplies [22] and in vending machines in health and social care institutions in Slovenia [26].

### Statistics

Descriptive statistics expressed as averages with standard deviation (SD) or percentages were used in data analysis. Additionally, percentages of beverages categorised with dietary traffic light labelling were shown, which served as overall quality indicator of the displays in hospitals, community health centres and nursing homes.

## Results

Altogether, 188 institutions were surveyed, of which 134 had at least one food/beverage vending machine on their premises. No vending machines were present in 7.7% hospitals (2 out of 26), 12.5% community health centres (8 out of 64) and 44.9% nursing homes (44 out of 98). From the total of 5625 products found in the vending machines, 54.2% (n=3046) products were beverages and were further investigated in this study. The analysis excluded products from non-standard vending machines, e.g. freshly squeezed orange juice or warm beverage vending machines.

When looking at beverages displayed in different types of institutions, 26.9% (n=819) beverages were from hospital vending machines, 36.8% (n=1120) from community health centre vending machines and 36.3% (n=1107) from vending machines placed in nursing homes. Beverages amounted to slightly more than half, i.e. 54.2% (n=3046) of the products in vending machines, which varies depending on the type of institution, with 51.7% in hospitals, 52.8% in community health centres and 57.4% in nursing homes.

**Fig. 1:**
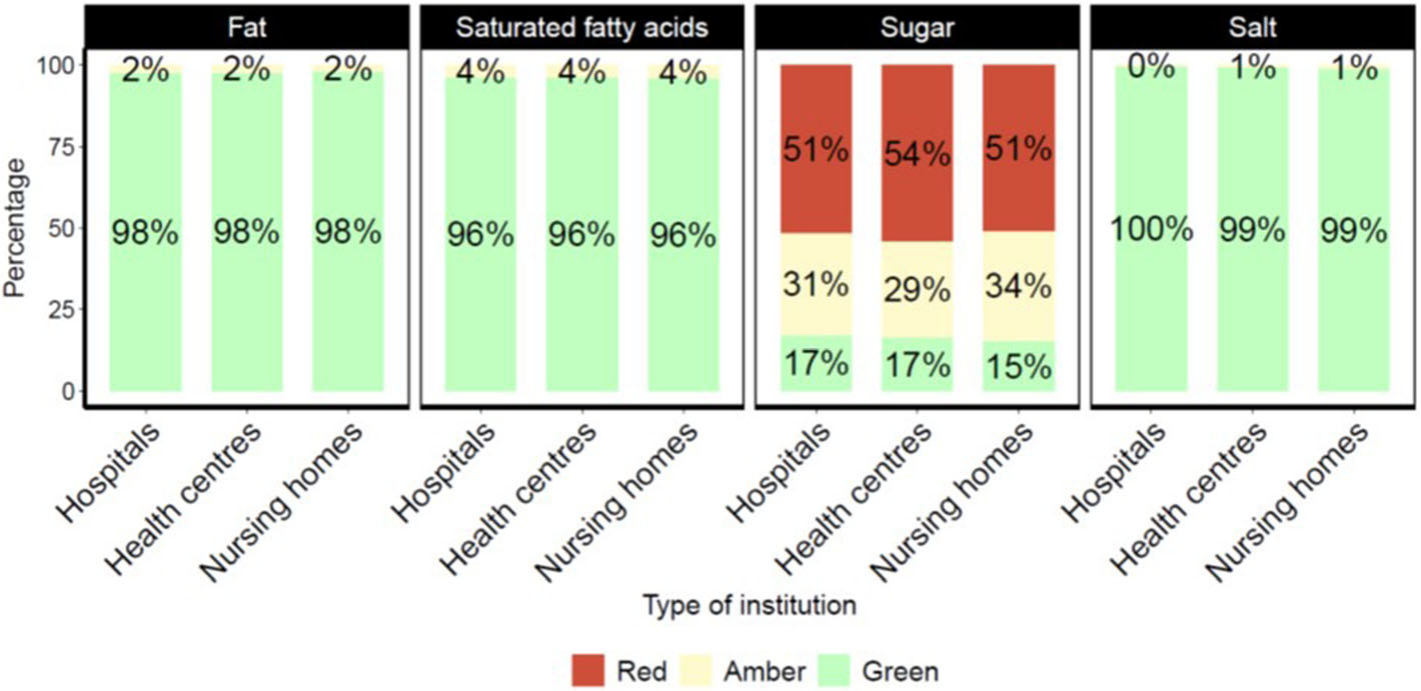
Percentage (%) of beverages in the vending machines in health and social care institutions categorised into green/amber/red (traffic light profiling)

Overall quality indicators for fat content, saturated fats, sugars and salts showed that the main problem in beverages sold in vending machines were sugars; considering traffic light profiling, between 51 and 54% of beverages fall into the red category. This is followed by the amber category regarding the sugar content (for 29–34% of beverages). Only 15 to 17% of beverages have been assigned a green category for the sugar content (Fig. 1).

As seen in Table 3, 82.3% out of 3046 beverages in vending machines were devoted to sugar-sweetened beverages. Smoothies accounted for only 0.4% and 100% juice for only 3.3% of beverages sold in vending machines. Out of 134 locations, smoothies were found at only five different locations, and 100% juices at only 51 different locations. Water accounted for only 13.7% of the beverages, available in vending machines. A more detailed overview of results where additional subcategories with average calories, sugar content and results of nutrient profiling using the FSANZ model are shown in Table 3.

**Table 3:** Average calories and sugar content of beverage categories with FSANZ model nutrient profiling

	Average from all health and social care institutions (n=188)					
	Total share in relation to all beverages listed [%]	Average calories [kcal in 100 mL]		Average sugar content [in 100 mL]		FSANZ
		Mean	SD	Mean	SD	(% less healthy products)
**Beverage categorisation**						
Soft drinks	75 (n=2286)	30.3	14.64	7.5	4.75	66.4
Waters	13.7 (n=417)	0	0	0	0	0.0
Coffee	3.9 (n=119)	66.3	3.96	8.7	0.66	99.2
Juices	3.7 (n=113)	42.2	17.58	8.6	3.42	14.2
Energy drinks	3.6 (n=111)	45.7	6.03	10.8	1.48	98.2
**Beverage subcategorisation**						
Non-carbonated beverages with sugar	30.3 (n=924)	33.6	14.37	7.9	2.13	87.0
Carbonated beverages with sugar	23.1 (n=704)	39.2	9.99	10.8	6.3	100.0
Sugar-tasting water	18.6 (n=568)	18	4.47	4	0.38	0.0
Mineral water	8 (n=243)	0	0	0	0	0.0
Water	5.7 (n=174)	0	0	0	0	0.0
Iced coffee	3.9 (n=119)	66.3	3.96	8.7	0.66	99.2
Energy drinks with sugar	3.6 (n=109)	46.5	0.34	11	0.11	100.0
100% natural juice	3.3 (n=102)	40.4	16.96	8.4	3.38	15.7
Carbonated drinks with no sugar and added aspartame/acesulfame-k sweetener	1.4 (n=43)	0.3	0.13	0	0	0.0
Water with taste but no sugar	1 (n=31)	2.3	0.16	0	0	0.0
Non-alcoholic beer	0.5 (n=16)	19.4	9.15	4.5	2.06	56.2
Smoothie	0.4 (n=11)	58.8	14.84	10.9	3.1	0.0
Energy drinks with no sugar and added aspartame/acesulfame-k sweetener	0.1 (n=2)	1.4	0	0	0	0.0

According to nutrient profiling using the FSANZ model, about 60% (n=1760) of all beverage front-face items available in vending machines can be considered as lower nutritional quality. Iced coffee and energy drinks present product categories with lower nutritional quality by FSANZ (99.2–98.2% of products), especially because of high sugar content and energy density, as well as absence of any beneficial nutrients. Among soft drinks, non-carbonated beverages contain slightly less sugar and less calories as carbonated beverages on average. Therefore, 13% of non-carbonated beverages, out of the whole sample, were evaluated as healthy by the FSANZ model, while another 87% and all 100% of carbonated beverages were evaluated as unhealthy. However, the minority of 100% natural juices are evaluated as less healthy (15.7%) because of high percent of fruit present and no added sugar, but still being a relatively high-energy product. Similarly, smoothies were evaluated as healthy due to their higher fruit content, despite the naturally present sugar. Smoothies and iced coffee with added sugar are standing out as products with the highest average calories per 100 ml, but a FSANZ evaluation is indicating major differences. While smoothies are high-calorie products at the expense of natural sugar present from fruits, iced coffee is considered high calorie because of added sugar (average 8.7 g/100 ml) and therefore 99.2% of iced coffee products are evaluated as less healthy. It should be noted that according to the FSANZ model, all non-sweetened beverages and beverages with added sweeteners were identified as healthy products (Table 3).

Also of note is that soft drinks offered in vending machines in health and social care institutions are displayed in 500-ml bottles in almost 60% of displays (Table 4).

**Table 4:** Size of packaging for beverages available in vending machines in health and social care institutions (n beverages= 3046)

Beverages category	Size of packaging [mL]	
	0.2–0.35 [%]	0.5 [%]
Soft drinks	16.11 (n=491)	58.93 (n=1795)
Waters	0 (n=0)	13.69 (n=417)
Coffee and tea	3.91 (n=119)	0 (n=0)
Juices	3.71 (n=113)	0 (n=0)
Energy drinks	3.09 (n=94)	0.56 (n=17)
**Total share [%]**	26.82 (n=817)	73.18 (n=2229)

## Discussion

When comparing beverage products from vending machines in different health and social care institution (hospital, community health centre, nursing home), the results do not significantly differ between the various institutions. We can estimate that the availability of beverages, as well as the nutritional quality of the beverages, is quite similar. In this study, we found that 56% of all beverage front-face items available from vending machines is of lower nutritional quality. This proportion is slightly higher when compared to the beverages available in the Slovenian food supply, where 50% was shown in a previous study [22]. A recent international comparison [23] showed considerable potential for improving nutritional quality for beverages. Slovenia was in the bottom third with lower nutritional quality for beverages and average sugar content of 7.5 g and 170kJ per 100 ml [23]. The average sugar content and average calories in beverages sold in vending machine are slightly lower when compared with an average sugar content of 6.7 g and 120 kJ per 100 ml in beverages sold in food stores. Beverages with no sugar and with non-sugar sweeteners are evaluated as healthier by FSANZ; however, this is in contradiction with the WHO nutrient profile model [27], which does not support the addition of non-sugar sweeteners to beverages. The most notable problem in displayed beverages is a very high free sugar content. This is not in line with the scientific opinions of several eminent professional associations that claim that children, adolescents and adults should drink water or unsweetened tea instead of sugar-sweetened beverages and natural juices [8–11]. WHO guidelines recommend adults and children to reduce their daily intake of free sugars to less than 10% (roughly 50 g) of total energy intake, while the European Society for Paediatric Gastroenterology Hepatology and Nutrition (ESPGHAN) and British recommendations (SACN) recommend less than 5% of total energy intake (3.5–9 g/day for children and adolescents aged 2–18 years). Compared to the results of our survey, the recommended daily intake of free sugars is nearly used when consuming only one average 500-ml carbonated beverage with free sugar. Although 100% natural juices seem like a healthy option, free sugar is naturally occurring and in liquid form (not added sugar) in those products, which, in addition to high intake, also affect the risk of developing obesity and non-communicable diseases [10]. When choosing one average sugar-sweetened beverage form a vending machine, containing approximately 140 kcal, the 5 to 7% of recommend daily calorie intake for an adult person and up to 12% of recommend daily calorie intake for small children [28] is already used.

Considering the increasingly popular vending machines and the trend of eating out [29–33], vending machines can be considered as an important environmental factor contributing to the obesity epidemic. Our results comply with the findings of other studies that snacks and drinks at vending machines located in various settings have limited healthy choices and high-energy and low-nutrient snacks are the first-choice options [1, 3, 4, 34–36]. In this context, vending machines represent a potential point of intervention for changing buyers’ eating habits to healthier choices [3]. Foods and beverages sold in vending machines are increasingly being scrutinized and addressed by federal policies. As a result, calls for local authorities to develop programs to promote healthier vending machines have been made overseas [8, 37–41]. The Slovenian School Nutrition Law [42] has prohibited vending machines in Slovenian primary and secondary schools since 2012. However, placing vending machines, as well as the nutritional values of available products in social and healthcare institutions, is not regulated by law. Thus, even those consumers who are aware of a healthy diet at the moment of choosing do not have the option of a healthy choice [36]. Healthier vending machine programs have already been implemented abroad in various settings such as hospitals [43], city parks [44], public buildings and government offices [45], and schools and university campuses [46–50]. Strategies that have already been used overseas mainly include enhancing healthier products on offer [43, 51–53] and changing prices and promoting healthier choices with posters, brands and stickers [3, 4, 54–57]. Since offering healthier options may be associated with shorter shelf life and higher product cost, an integrated approach is needed. Already available technical adaptations of vending machines as a refrigerated food vending may offer opportunity to have fresh healthy foods in vending, rather than having to rely on the limited range of pre-packaged items. In order to ensure acceptable product prices, the industry should be encouraged for product reformulation towards healthier options [58]. The general opinion that healthy food product is also more expensive is not necessarily correct [59]. In fact, research at vending machines across university campuses show that the difference between the prices of healthier and less healthy foods and beverages was $0.72 and $0.16, respectively, but these differences were not statistically significant [60]. Also, several countries in Europe have already introduced health-related taxes on specific foods with the objective of influencing what people are buying [61]. Overall, health should be prioritised over sales profit, and low-earning machines can be moved to high-traffic areas [58].

Another concern that should be highlighted is that the majority of beverages are displayed in plastic packaging, which contributes to the high carbon footprint of bottled water [62, 63]. Bottled water in Slovenia is also incomparably expensive compared with tap water [64]. Used once, the plastic packaging ends up at the waste collection centre (or even in nature), where in Slovenia, only 26% of plastics are recycled, mostly in downgrading processes [64, 65]. Approximately 36% of plastic packaging is re-used for energy by incineration [65], where toxic by-products, such as dioxins, are produced. Dioxins are toxic substances that show harmful effects on the skin and the immune system, are reproductive toxic and teratogenic, interfere with hormonal balance and are carcinogenic [66]. Plastics will, under environmental impacts, slowly degrade into microplastics, for which bioaccumulation and biomagnification processes through the food chain can cause concern for human health [67, 68]. As selling beverages in vending machines require packaging, the disadvantages of plastics could be addressed by introducing alternative materials for packaging, such as bioplastics [69], glass, metals or paper and paperboard [70, 71].

## Conclusion

Based on the above findings, we propose that regulatory guidelines should be included in the tender conditions for vending machines in health and social care institutions to ensure healthy food and beverage choices.

## Data Availability

Not applicable.
